# Old Habits Die Hard: A Nationwide Utilization Study of Short-Acting Nifedipine in Taiwan

**DOI:** 10.1371/journal.pone.0091858

**Published:** 2014-03-17

**Authors:** Chia-Lin Chou, Chia-Yu Chou, Chia-Chen Hsu, Yueh-Ching Chou, Tzeng-Ji Chen, Li-Fang Chou

**Affiliations:** 1 Department of Pharmacy, Taipei Veterans General Hospital, Taipei, Taiwan; 2 Department of Critical Care Medicine, Taipei Veterans General Hospital, Taipei, Taiwan; 3 Department and Institute of Pharmacology, National Yang-Ming University, Taipei, Taiwan; 4 College of Pharmacy, Taipei Medical University, Taipei, Taiwan; 5 Department of Internal Medicine, School of Medicine, National Defense Medical Center, Taipei, Taiwan; 6 Department of Medicine, Tzu Chi University, Hualian, Taiwan; 7 Institute of Hospital and Health Care Administration, School of Medicine, National Yang-Ming University, Taipei, Taiwan; 8 Department of Family Medicine, Taipei Veterans General Hospital, Taipei, Taiwan; 9 Department of Public Finance, National Chengchi University, Taipei, Taiwan; National Taiwan University, Taiwan

## Abstract

**Background:**

To investigate the nationwide trend of ambulatory prescriptions of short-acting nifedipine on a PRN (pro re nata) order over a fifteen-year period in Taiwan.

**Methods:**

The systematic sampling claims datasets (0.2% sampling ratio) of ambulatory care visits within Taiwan's National Health Insurance from 1997 to 2011 were analyzed. The prescriptions of short-acting capsule-form nifedipine on a PRN order were stratified by the patient's age, the prescribing physician's specialty, and the setting of healthcare facility for each year.

**Results:**

During the study period, 8,189,681 visits were analyzed. While the utilization rate of calcium channel blockers changed with time from 2.8% (13,767/489,636) in 1997 to 5.1% (31,349/614,719) in 2011, that of short-acting nifedipine were from 1.0% (n = 5,070) to 0.2% (n = 1,246). However, short-acting capsule-form nifedipine on a PRN order still existed (from 447 prescriptions in 1997 to 784 in 2011). More than one half of these PRN nifedipines were prescribed by the internists and to the elderly patients; almost four-fifths of PRN nifedipines were prescribed during non-emergent consultations.

**Conclusion:**

The physicians in Taiwan still had the habit of prescribing short-acting nifedipines for PRN use. The reason for such practices and the impact on patients' health deserve attention.

## Introduction

Calcium channel blockers (CCBs) are antihypertensive agents widely prescribed for decades [1], [2]. CCBs are heterogeneous, not only in ingredient and formulation, but also in indication [3]. Short-acting nifedipine used to be a common treatment option for severe hypertension [4]. However, several studies have reported the serious outcome from rapid fluctuation of blood pressure after sublingual use of nifedipine [5], [6]. Although the Food and Drug Administration (FDA) in the USA has announced the warning about the use of short-acting nifedipine in the management of hypertension crisis in 1996 [7], such a medical practice is still observed worldwide [8], [9]. A long-term observational study of short-acting nifedipine use has been seldom reported.

The aim of the current study was to investigate the trend of ambulatory nifedipine utilization in Taiwan according to the nationwide insurance claims from 1997 to 2011. Special attention would be paid to the extent and situations of prescribing short-acting nifedipine on a PRN (pro re nata) order.

## Materials and Methods

### Data source

We obtained the sampling claims datasets of ambulatory care visits at clinics of Western medicine 1997–2011 (CD{1997..2011}0.DAT and OO{1997..2011}0.DAT) from the National Health Research Institutes in Miaoli, Taiwan, which has managed the whole archived claims of the National Health Insurance (NHI) in Taiwan through the project of the National Health Insurance Research Database (NHIRD) (http://www.nhri.org.tw/nhird/). The NHI in Taiwan has started as a universal health insurance program since 1995 and covered nearly all inhabitants (23,198,664 beneficiaries at the end of 2011, equivalent to a coverage rate of 99.9%) [10]. The two files of linked visit and prescription records in each year were extracted from the complete database of ambulatory care claims, including visits to emergency departments but excluding services for dental and traditional Chinese medicine, with a sampling ratio of 0.2%. The structure and contents of the NHI claim files had been described in some earlier papers [11]–[13]. Any identification data about patients and healthcare facilities in the NHIRD datasets have been encrypted to protect privacy. Researchers who wish to access NHIRD datasets must sign a user agreement form indicating they will obey related regulations and acknowledge the NHIRD in their publications.

We also downloaded the master file of 16,948 approved drug items of Western medicine in Taiwan from the web site of the Bureau of National Health Insurance (http://www.nhi.gov.tw/, accessed 10 July 2013).

### Study design

The conduct of the study had been approved by the institutional review board of Taipei Veterans General Hospital, Taipei, Taiwan (2013-01-005E).

At first, we calculated the prescriptions with antihypertensive agents in Taiwan in each year to have a general view. The antihypertensive agents defined in our study were all drug items belonging to the groups C02 (antihypertensives), C03 (diuretics), C07 (beta blocking agents), C08 (calcium channel blockers), and C09 (agents acting on the renin-angiotensin system) of the Anatomical Therapeutic Chemical (ATC) classification system version 2013 (http://www.whocc.no/atcddd/indexdatabase/index.php, accessed 10 July 2013). Totally, 1,472 items of antihypertensive agents had been registered in the NHI drug file since 1995, including 314 items of CCBs.

Then, our analysis was concentrated on the nifedipine prescriptions, stratified by individual formulation. In the NHI drug file, there were 86 items of nifedipine, including 17 items of sustain-released nifedipine, 7 items of short-acting nifedipine tablet, and 62 items of nifedipine capsule.

Because the route of medication was not contained in the NHIRD datasets, we identified the nifedipines prescribed as a PRN order from the field of medication frequency of the prescription files. These nifedipine prescriptions were further stratified by the patient's age, the prescribing physician's specialty, and the setting of healthcare facility.

### Data processing

Descriptive data were presented. The computation was undertaken with the Perl programming language (version 5.18.0, http://www.perl.org/). Besides, we used the chi-square trend test (linear-by-linear association) to detect the temporal (yearly) patterns of CCB use among the ambulatory visits and among the prescriptions with antihypertensive agents. A *p* value <0.05 (two-sided) was regarded as statistically significant. The statistical analyses were performed with the SPSS software (Release 17.0 for Windows, SPSS Inc., Chicago, Illinois).

## Results

In the visit-based sampling datasets of our current study, the number of records increased from 489,636 in 1997 to 614,719 in 2011 (Table 1). The annual visits with prescriptions of antihypertensive agents also increased from 6.7% (32,629/489,636) in 1997 to 10.6% (65,134/614,719) in 2011 (*p*<0.001 in linear-by-linear association). The CCBs had remained as the popular antihypertensive agents. Not only increased the share of CCBs in prescriptions with antihypertensive agents annually from 42.2% (13,767/32,629) in 1997 to 48.1% (31,349/65,134) in 2011 (*p*<0.001), but also their share in all visits had a continuous growth from 2.8% (13,767/489,636) in 1997 to 5.1% (31,349/614,719) in 2011 (*p*<0.001).

**Table 1:** Characteristics of ambulatory visits in Taiwan, 1997–2011 (0.2% sampling).

	1997	1998	1999	2000	2001	2002	2003	2004	2005	2006	2007	2008	2009	2010	2011
All visits	489,636	520,757	539,661	526,693	521,938	527,098	516,546	561,772	567,678	538,703	548,600	550,782	576,416	588,682	614,719
Visits with antihypertensive agents	32,629	38,861	42,521	40,979	44,293	46,356	46,832	52,948	54,566	55,252	57,494	59,665	61,136	63,232	65,134
Visits with CCBs^a^	13,767	16,676	18,173	17,445	19,392	20,745	20,955	24,289	25,335	26,278	28,226	29,739	30,071	30,784	31,349
Visits with nifedipine	6,154	6,940	6,835	5,610	5,570	5,184	4,735	4,898	4,821	4,743	4,684	4,543	4,275	4,313	4,142
Visits with short-acting nifedipine	5,070	5,495	5,159	3,812	3,491	2,920	2,525	2,359	2,083	1,791	1,579	1,432	1,352	1,335	1,246
Visits with short-acting capsule-form nifedipine	4,940	5,352	5,042	3,746	3,460	2,918	2,524	2,359	2,083	1,791	1,579	1,432	1,352	1,335	1,246
Visits with short-acting capsule-form nifedipine PRN^b^ use	447	530	656	634	689	667	715	825	761	703	717	670	710	746	784
No. of patients	445	529	653	631	689	666	713	822	760	702	716	667	710	746	783
No. of prescribers	379	432	527	530	580	571	594	676	640	595	603	572	593	607	647

a CCBs: calcium channel blockers.

b PRN: pro re nata.

Despite the growth of CCBs, there was a decreasing trend of nifedipine utilization in prescriptions with antihypertensive agents from 18.9% (6,154/32,629) in 1997 to 6.4% (4,142/65,134) in 2011 (*p*<0.001 in linear-by-linear association). During the study period, 1.3% (10,254/761,898) of prescriptions with antihypertensive agents and 2.9% (10,254/353,224) of prescriptions with CCBs contained a PRN order of short-acting capsule-form nifedipines. While the use of short-acting capsule-form nifedipines significantly decreased, the number of PRN orders did not decrease accordingly among visits with short-acting capsule-form nifedipines (447 in 1997 and 784 in 2011).

A total of 10,232 patients have ever received short-acting capsule-form nifedipines on a PRN order during 1997–2011 (445 in 1997 and 783 in 2011). More than one half of these nifedipines on a PRN order were prescribed by the internists and to the elderly patients; almost four-fifths of these nifedipines on a PRN order were prescribed during non-emergent consultations (Table 2).

**Table 2:** Ambulatory prescriptions with short-acting nifedipine as a PRN (pro re nata) order in Taiwan, 1997–2011 (0.2% sampling).

	1997	1998	1999	2000	2001	2002	2003	2004	2005	2006	2007	2008	2009	2010	2011
By patients' age (year)															
65 and plus	226	282	325	311	353	353	384	446	438	405	395	393	412	387	448
40–64	204	224	307	298	307	285	295	345	297	270	298	248	271	317	305
18–39	15	23	22	25	29	29	36	34	25	28	24	29	27	41	31
0–17	1		2						1					1	
Unknown	1	1													
By physicians' specialty															
General practice	117	167	199	190	204	174	193	230	217	194	142	127	160	128	144
Cardiology	8	17	40	56	64	74	89	114	93	119	119	117	122	145	151
Internal medicine except cardiology	251	288	336	278	296	265	249	302	274	221	268	232	203	221	251
Others	71	58	81	110	125	154	184	179	177	169	188	194	225	252	238
By setting															
Emergency department	94	101	115	141	142	159	146	157	178	157	153	137	165	191	181
Others	353	429	541	493	547	508	569	668	583	546	564	533	545	555	603
Total	447	530	656	634	689	667	715	825	761	703	717	670	710	746	784

## Discussion

In this current study, we used a nationwide sampling database with a sampling ratio of 0.2%, so one beneficiary was rarely sampled more than twice. Based on the nationally sampled datasets, our study revealed that the CCBs continued to be the popular antihypertensive agents in Taiwan from 1997 to 2011. Besides, the short-acting nifedipines were still in use on the market. Although the share of short-acting nifedipines in all prescriptions with antihypertensive agents decreased with time, short-acting nifedipines on a PRN order did not vanish in clinical practice.

The continuous popularity of CCBs in Taiwan was due to increasing use of newer-generations CCBs instead of medication persistency. The CCB utilization in Taiwan did not seem to be influenced by the worldwide CCB controversy starting in 1995 [14]. Unlike some utilization studies of CCBs in other countries [1], [15], [16], the lack of prescription data prior to 1997 in Taiwan precluded us from conducting a before-and-after analysis to understand the impact of the CCB controversy. On the other side, although we could not truly identify the sublingual nifedipine for treating acute hypertension in our study, the PRN use of short-acting nifedipine in Taiwan was not influenced by the disapproval of the Food and Drug Administration in the USA in 1995 as to the sublingual use of nifedipine either [17].

Patients of different ages might respond to the sublingual nifedipine differently. While even a low dose of sublingual nifedipine could induce risk of myocardial or cerebral ischemia in elderly patients [8], [18], the short-acting nifedipine for treating acute hypertension in children was still recommended by some authors [19]–[22]. Because the prevalence of hypertension and hypertensive emergencies was highly related to age, we could hardly observe the PRN use of short-acting nifedipine in children in our study. Instead, the predominant use by the elderly in Taiwan deserved concern. Another concern was about the setting in which short-acting nifedipine on a PRN order was prescribed. While the adverse reaction could be more easily monitored and managed at the hospitals, the take-home medication would let the patients get into danger unsupervised.

Hypertension is a chronic disease and the current guidelines recommend a long-term maintenance of normal blood pressure (BP) level. For severe BP elevation, the treatment goal is to reduce 20–25% of mean BP within the first hour and below 160/100–110 mmHg within the next 2–6 hours. The rapid and excessive reduction of BP from effect of short-acting nifedipine should be avoided to prevent organ ischemia [23], [24]. In Beers Criteria, the use of immediately-released nifedipine is also strongly considered as the potentially inappropriate medication and should be avoided for the elderly [25]. Despite these recommendations, Grise et al. reported that the rate of change in BP was greater at real-world emergency department setting than the guideline recommended [26]. In our study, physicians remained accustomed to the habit of prescribing short-acting nifedipine as a PRN order both in emergency and outpatient department, and the predominant users were the elderly. The reason that physicians still prescribe inappropriate drugs for patients is unclear. McGlynn et al. have reported the gap between physicians' knowledge and their clinical practice [27]. Prescriptions of short-acting nifedipine for hypertensive patients might successfully vanish from intervention with educational efforts [9].

Our descriptive study has some limitations. We had not yet studied the risks from inadequate dosing [28], interacting drugs [29], and co-morbidity. Further stratification of the results by patients' or physicians' characteristics was not performed because of the relatively small number of short-acting nifedipine PRN use in our sampling datasets. The outcome of the PRN use of short-acting nifedipine was also beyond the scope of our study of visit-based sampling datasets. Besides, the relative factors or prescribing habits of individual physicians could not be studied from the visit-based sampling datasets [30]. Furthermore, the actual use of a PRN medication at ambulatory setting could hardly be confirmed in the prescription database. Neither could the PRN use of accumulated drugs at home be known.

## Conclusions

In conclusion, the physicians in Taiwan still had the inadequate habit of prescribing short-acting nifedipines both on regular use and on PRN use. The reason for such practices and the impact on patients' health deserve attention.
